# Influence of Soy Lecithin and Sodium Caseinate on The Stability and *in vitro* Bioaccessibility of Lycopene Nanodispersion

**DOI:** 10.17113/ftb.61.01.23.7538

**Published:** 2023-03

**Authors:** Nor Shariffa Yussof, Tan Chin Ping, Tan Tai Boon, Uthumporn Utra, Muhammad Ezzudin Ramli

**Affiliations:** 1 Department of Food Technology, School of Industrial Technology, Building G07, Persiaran Sains, Universiti Sains Malaysia, Jalan Sungai 2, 11800 USM Penang, Malaysia; 2 Department of Food Technology, Faculty of Food Science and Technology, Universiti Putra Malaysia, Jalan Universiti 1, 43400 UPM Serdang, Selangor, Malaysia

**Keywords:** lycopene, nanodispersion, bioaccessibility, sodium caseinate, lecithin, emulsifier

## Abstract

**Research background:**

Various approaches have been used to present functional lipids including lycopene in a palatable food form to consumers. However, being highly hydrophobic, lycopene is insoluble in aqueous systems and has a limited bioavailability in the body. Lycopene nanodispersion is expected to improve the properties of lycopene, but its stability and bioaccessibility are also affected by emulsifier type and environmental conditions such as pH, ionic strength and temperature.

**Experimental approach:**

The influence of soy lecithin, sodium caseinate and soy lecithin/sodium caseinate at 1:1 ratio on the physicochemical properties and stability of lycopene nanodispersion prepared using the emulsification-evaporation methods before and after treatment at different pH, ionic strength and temperature were investigated. The *in vitro* bioaccessibility of the nanodispersions was also studied.

**Results and conclusion:**

Under neutral pH conditions, nanodispersion stabilized with soy lecithin had the highest physical stability and the smallest particle size (78 nm), the lowest polydispersity index (PDI) value (0.180) and highest zeta potential (-64 mV) but the lowest lycopene concentration (1.826 mg/100 mL). Conversely, nanodispersion stabilized with sodium caseinate had the lowest physical stability. Combining the soy lecithin with sodium caseinate at 1:1 ratio resulted in a physically stable lycopene nanodispersion with the highest lycopene concentration (2.656 mg/100 mL). The lycopene nanodispersion produced by soy lecithin also had high physical stability under different pH range (pH=2-8) where the particle size, PDI and zeta potential remained fairly consistent. The nanodispersion containing sodium caseinate was unstable and droplet aggregation occurred when the pH was reduced close to the isoelectric point of sodium caseinate (pH=4-5). The particle size and PDI value of nanodispersion stabilized with soy lecithin and sodium caseinate mixture increased sharply when the NaCl concentration increased above 100 mM, while the soy lecithin and sodium caseinate counterparts were more stable. All of the nanodispersions showed good stability with respect to temperature changes (30–100 °C) except for the one stabilized by sodium caseinate, which exhibited an increased particle size when heated to above 60 °C. The combination of soy lecithin and sodium caseinate was found to increase the bioaccessibility of the lycopene nanodispersion. The physicochemical properties, stability and extent of the lycopene nanodispersion digestion highly depend on the emulsifier type.

**Novelty and scientific contribution:**

Producing a nanodispersion is considered one of the best ways to overcome the poor water solubility, stability and bioavailability issues of lycopene. Currently, studies related to lycopene-fortified delivery systems, particularly in the form of nanodispersion, are still limited. The information obtained on the physicochemical properties, stability and bioaccessibility of lycopene nanodispersion is useful for the development of an effective delivery system for various functional lipids.

## INTRODUCTION

An emulsifier has a major role in the food industry, especially in the production of dairy products, baked goods and beverages. In recent years, in-depth studies of the use of various emulsifiers in the production of nanoemulsions or nanodispersions as a delivery system for poorly water-soluble compounds (such as carotenoids, antioxidants and flavour compounds) for food application have been extensively conducted. Many studies have shown that nanoemulsions can improve the solubility of hydrophobic compounds, increase the absorption rates, enhance the bioavailability and improve physical stability against destabilization processes (*i.e.* flocculation, creaming, coalescence, and Ostwald ripening) and environmental stresses (*1*-*3*). However, the characteristics and stability of the resulting nanoemulsion also depend on the type of emulsifier used.

Soy lecithin and sodium caseinate are examples of food-grade emulsifying agents that are widely used in the food industry. The sodium caseinate molecules are able to adsorb, unfold and spread at the droplet surface to form a thick coating layer that generates strong steric stabilization. Soy lecithin is a phospholipid containing up to 29–46% phosphatidylcholine, 21–34% phosphatidylethanolamine and 13–21% phosphatidylinositol (*4*). It also contains minor amounts of phosphatidic acid, phosphatidylserine, and non-phospholipids components such as triglycerides, free fatty acids, and carbohydrates. Soy lecithin contributes to a good stability of an emulsion by adsorbing rapidly onto the droplet surface, efficiently reducing the interfacial tension between the oil and water phases, and imparting a high negative net charge to the emulsion, which results in high repulsive force interactions against the destabilization processes. One of the most common approaches to produce an emulsion with the desired characteristics is by combining different types of emulsifier to stabilize the emulsion such as chitosan (*5*); starch (*6*), protein (*7*), sodium caseinate and soy lecithin (*8*).

The use of various processing methods and conditions as well as numerous ingredients in food processing may expose foods to various environmental conditions such as pH, ionic strengths and temperature, thus affecting the emulsifying properties of food emulsifiers.

Bioaccessibility should also be taken into consideration when designing food products as it is affected by the structure and particle size of the compounds, the nature of the food matrix, and its interaction with other food constituents (*9*). An evaluation of bioaccessibility is sufficient to estimate the bioavailability of carotenoids such as *in vitro* digestion, which is considered rapid, inexpensive, reproducible and reliable method (*10*). Many different *in vitro* models have been developed based on the studied food or carotenoids, but the general steps are largely similar, *i.e.* simulation of the digestion conditions in the gastrointestinal tract followed by quantification of the carotenoid fraction released from the food matrix and incorporated into the bile salt micelles *via* high-performance liquid chromatography (HPLC) (*11*, *12*).

Lycopene is a natural carotenoid pigment consisting of a straight hydrocarbon chain with 11 conjugated double bonds and 2 non-conjugated double bonds that are susceptible to degradation, oxidation and/or *trans-cis* isomerization under light, oxygen, heat and acidic or alkaline conditions (*13*). The presence of unsaturated double bonds in the lycopene demonstrated several biological activities such as antioxidant (*14*), immunoregulation (*15*) and anticancer (*16*) as preventive effects against nerval degenerative diseases (*17*), breast cancer (*18*) and cardiovascular diseases (*19*).

In this study, lycopene nanodispersions were prepared by using different emulsifier types, namely soy lecithin, sodium caseinate, and a combination of soy lecithin and sodium caseinate at 1:1 ratio, using emulsification and evaporation method. The effects of emulsifier types and environmental conditions (pH, ionic strength and temperature) on the particle size, polydispersity index, zeta potential and lycopene concentration of the nanodispersions were determined. The *in vitro* bioaccessibility of the lycopene nanodispersions was also investigated using a 2-phase static *in vitro* gastrointestinal digestion model.

## MATERIALS AND METHODS

### Materials

Lycopene (95%) was purchased from Shaanxi Jinjiankang Biological Technology Co., Ltd. (Xi’an, PR China). Sodium caseinate, soy lecithin, sodium chloride and sodium hydroxide were purchased from Fisher Scientific (Leicester, UK). Ascorbic acid solution, pancreatin from porcine pancreas, bile extract, pyrogallol, dl-α-tocopherol, potassium chloride, calcium chloride dihydrate, potassium dihydrogenphosphate, magnesium chloride hexahydrate and sodium hydrogencarbonate were purchased from Sigma-Aldrich, Merck (St. Louis, MO, USA). The deionized water for nanodispersion preparation was produced using a Sartorius Stedim Biotech Arium 611DI system (Göttingen, Germany).

#### Preparing a lycopene nanodispersion

Different types of emulsifiers, namely soy lecithin, sodium caseinate, and a combination of soy lecithin and sodium caseinate in the ratio of 1:1 were used to prepare lycopene nanodispersions. All samples were prepared at neutral pH. For the mixture of soy lecithin and sodium caseinate, both emulsifiers were mixed before the emulsification process. Their 1:1 ratio was chosen as it showed the best synergistic effects on the lycopene nanodispersion with the smallest particle size and the highest zeta potential during our preliminary study.

#### Pre-emulsification step

Lycopene powder extract was dissolved in dichloromethane using a magnetic stirrer (MR Hei-Tec; Heidolph, Schwabach, Germany) at room temperature (25 °C) to form an organic phase. The aqueous phase consisting of an emulsifier dissolved in deionized water was also prepared using magnetic stirring. Coarse oil-in-water emulsion was obtained by mixing the organic phase with the aqueous phase at a ratio of *ɸ*=1:9 in a rotor-stator homogenizer (model L4R; Silverson, Chesham, UK) at 523.5 rad/s for 5 min.

#### Emulsification in a high-pressure homogenizer

The resulting coarse pre-emulsion was passed through a high-pressure homogenizer (Panda Plus 2000; GEA Niro Soavi, Parma, Italy) for 2 homogenization stages at 30 MPa for 2 cycles. The dichloromethane in the fine emulsion was removed by using a rotary evaporator (Eyela NE-1001; Tokya Rikakikai Co. Ltd, Tokyo, Japan) under a reduced pressure of 25 kPa for 20 min at 40 °C and 10.5 rad/s.

### Effect of environmental changes

#### pH

The influence of pH (pH=2-8) on the characteristics of lycopene nanodispersions were measured using a Zetasizer Nano-ZS instrument (Malvern Instruments Ltd., Malvern, UK) coupled with an automatic titrator unit (autotitrator MPT-2). The titrations were done automatically in a plastic sample container, which was connected through a capillary system and a peristaltic pump with a folded capillary zeta potential cell (DTS 1060; Malvern Instruments Ltd).

#### Ionic strength

The effects of ionic strength on the characteristics of lycopene nanodispersion were measured by adding appropriate amounts of 1 M NaCl solution into the freshly prepared samples to obtain final NaCl concentrations of 0, 100, 200, 300, 400 and 500 mM. The diluted samples were stored in glass amber bottles at ambient temperature (25 °C) for 24 h prior to analysis. The experiments were carried out under low light conditions.

#### Temperature

The effects of temperature on the stability of lycopene nanodispersions were studied by incubating the freshly prepared samples stored in amber bottles in a water bath (WBU 45; Waterbath, Memmert, Schwabach, Germany) set at 30, 40, 60, 80 and 100 °C for 1 h. Then, the samples were kept in a dark place at ambient temperature (25 °C) for 24 h prior to analysis. The experiments were carried out under low light conditions.

### In vitro bioaccessibility

The bioaccessibility of the lycopene nanodispersion stabilized by different emulsifier types was measured by the 2-phase static *in vitro* model simulating the digestion process in the stomach (gastric phase) and upper part of the small intestine (duodenum) as described by Ha *et al*. (*20*) with minor modifications. The oral phase was not included in the model because it does not have a significant impact on the bioaccessibility of the lycopene nanodispersion, as the main digestive process that occurs in the mouth is starch digestion by amylase, and the lycopene nanodispersions do not contain starch. Furthermore, lycopene is categorized as a lipid compound, and lipid digestion occurs in the stomach and small intestine (*21*). Digestion in the large intestine was not studied because most of the chemical digestion and nutrient absorption takes place in the small intestine (*22*). The bioaccessibility of the prepared lycopene nanodispersion was evaluated by measuring the lycopene concentration released from the food matrix (lycopene in the digesta supernatant) and the lycopene concentration incorporated into micelles.

To simulate the first half of the stomach phase, 5 mL of NaCl/ascorbic acid solution (0.9% (*m*/*V*) NaCl and 0.1% (*m*/*V*) ascorbic acid) and 5 mL of electrolyte solution (in g/L: NaCl 3, KCl 1.062, CaCl_2_·2H_2_O 1.468, KH_2_PO_4_ 0.470 and MgCl_2_·6H_2_O 0.740) were added to the sample solution (10 mL). The pH was adjusted to pH=4 by adding 1 M HCl. Then, 5 mL of pepsin solution were added to the mixture to prepare the simulated stomach conditions. The second half of the preparation of the simulated stomach conditions included reducing the pH to pH=2 by adding 1 M HCl. The mixtures were incubated in a water bath shaken at 26.175 rad/s for 30 min at 37 °C.

To simulate the small intestinal phase, the pH of the digesta from the previous simulated stomach phase was increased to pH=6.9 by adding NaHCO_3_. Then, 3 mL of pancreatin/bile extract solution (0.4% (*m*/*V*) pancreatin from porcine pancreas, 2.5% (*m*/*V*) bile extract, 0.5% (*m*/*V*) pyrogallol and 1% (*m*/*V*) dl-α-tocopherol) was added, and the mixture was incubated in a water bath shaken at 26.175 rad/s for 2 h at 37 °C.

To measure the lycopene concentration released from the food matrix, the *in vitro* digesta solution was centrifuged (centrifuge model 4000; Kubota Corporation, Osaka, Japan) at 5000×*g* for 15 min. The supernatant was vacuum-filtered, and the lycopene concentration in the supernatant was measured using HPLC. The concentration of lycopene incorporated in the bile salt micelles was measured by extracting the lycopene from the solution of the *in vitro* digesta as follows: 2 mL of the solution of the *in vitro* digesta was added to 4 mL of *φ*(hexane,acetone, ethanol)=2:1:1 mixture containing 0.1% butylated hydroxytoluene (BHT). Hexane (non-polar) was used to dissolve the lycopene, whereas acetone and ethanol (polar solvents) were used to remove water-soluble compounds from the samples and to obtain phase separation. The mixture of hexane, acetone and ethanol had previously been used to optimize lycopene extraction, particularly from tomato products (*9*). BHT was added as an antioxidant. NaCl solution (1 mL of *w*=25%) was added, and the mixture was vortexed for 10 min. The added NaCl was responsible for increasing the polarity of the polar phase and improving the separation between the polar and non-polar phases. The mixture was centrifuged at 5000×*g* for 10 min, and the organic phase in the upper layer was collected. The lycopene concentration in the organic phase was measured by HPLC.

The *in vitro* bioaccessibility was calculated using the following formula:

Bioaccessibility=[(*γ*_r_+*γ*_m_)/*γ*_i_]·100 /1/

where *γ*_r_ is the concentration of lycopene released from the food matrix (mg/mL), *γ*_m_ is the concentration of lycopene in micelles (mg/mL), and *γ*_i_ is the initial lycopene concentration (mg/mL).

### Characterizing the lycopene nanodispersion

#### Particle size and polydispersity index

The particle size and polydispersity index (PDI) of lycopene nanodispersions were determined using a dynamic light scattering device (Zetasizer Nano-ZS; Malvern Instruments). The particle size and PDI were measured at 25 °C under low light conditions. The mean particle diameters are reported as Z-average diameters (the scattering intensity-weighted mean diameter) in nm.

#### Zeta potential

The stability of the nanodispersions was analyzed by determining the net electrical charge using Zetasizer Nano-ZS (Malvern). A volume of 1 mL of freshly prepared lycopene nanodispersion was injected into disposable folded capillary cells (DTS 1060) and placed in the cell holder. The sample was then equilibrated at 25 °C for 120 s before measurement. The analysis was performed under low light conditions.

#### Determining the lycopene concentration

An aliquot of lycopene nanodispersion (1 mL) was mixed with 2 mL of *V*(hexane):*V*(acetone)= 2:1 and vortexed at 104.7 rad/s for 5 min. Then, the sample was centrifuged at 800×*g* for 10 min. The hexane upper layer containing lycopene was collected and the extraction method was repeated once again. The lycopene in the hexane layer was collected and an aliquot of 1 mL was then filtered through a 0.45-µm pore membrane filter. A volume of 20 µL of the filtrate were injected into an HPLC system (Waters e2695 HPLC separation modules equipped with a Waters 2489 UV-Vis detector; Waters, Milford, MA, USA). HPLC analysis was performed with a Waters liquid chromatography system equipped with a diode array detector and a Nova-Pak C18 (3.9 mm×300 mm) Waters HPLC column with an isocratic mobile phase consisting of 15% tetrahydrofuran, 30% acetonitrile and 55% methanol at 1 mL/min and temperature of 30 °C. Detection was performed at 472 nm. All steps were performed under subdued light.

### Statistical analysis

All measurements taken from freshly prepared samples are expressed as mean value±standard deviation (S.D.) of triplicate determinations (*N*=3). The data were analyzed with SPSS v. 12.0 software (*23*). Duncan’s multiple range test was used to compare significant differences between sample mean values at a 5% significance level (p<0.05).

## RESULTS AND DISCUSSION

### Influence of emulsifier type

In this study, the physical and chemical stability of lycopene nanodispersions stabilized by soy lecithin, sodium caseinate and their mixture was investigated by evaluating the particle size, polydispersity index (PDI), zeta potential and lycopene concentration of the freshly prepared sample. Based on the results shown in Table 1, all three emulsifiers were capable of producing lycopene nanodispersion at a nanoscale, whereby soy lecithin had the smallest mean particle size (78 nm), followed by the mixture of soy lecithin and sodium caseinate (91 nm) and sodium caseinate (159 nm). The soy lecithin-stabilized nanodispersion had the lowest PDI value, indicating the narrowest size distribution. The sodium caseinate-stabilized nanodispersion on the other hand exhibited the highest PDI value, demonstrating the broadest and less homogenous size distribution. The small particle size and low PDI value in a soy lecithin-stabilized nanodispersion were attributed to its small molecular mass and the ability to adsorb rapidly onto the droplet surface. Soy lecithin is a naturally occurring amphiphilic surfactant that is able to adsorb at the oil-water interface and reduce the interfacial tension between the oil and water. Its hydrophilic head group comprising phosphatidic acid, phosphatidylcholine, phosphatidylethanolamine, phosphatidylinositol or phosphatidylserine attached to the phosphate groups is attracted to water, while its hydrophobic tail group consisting of two fatty acids (*24*, *25*) is attracted to the oil phase. During the homogenization, soy lecithin is adsorbed rapidly at the oil-water interface and it forms a protective layer that prevents droplet aggregation and coalescence. The hydrophilic head group of lecithin, which can be anionic (phosphatidylinositol) or zwitterionic (phosphatidylcholine and phosphatidylethanolamine), also helps to prevent droplet coalescence by providing a negative surface charge to the dispersed droplets keeping them small in size.

**Table 1:** Particle size, polydispersity index (PDI), zeta potential and lycopene concentration of lycopene nanodispersion

Sample	*d*(particle)/nm	PDI	*ζ*/mV	*γ*(lycopene/(mg/100 mL)
Soy lecithin (SL)	(78.0±0.9)^a^	(0.180±0.008)^a^	(-64.0±0.2)^a^	(1.8±0.1)^c^
Sodium caseinate (SC)	(159.0±1.2)^b^	(0.41±0.01)^c^	(-45.0±0.1)^c^	(2.51±0.02)^b^
SL/SC	(91.0±0.5)^c^	(0.32±0.03)^b^	(-48.0±0.2)^b^	(2.66±0.05)^a^

The results are expressed as mean value±S.D. (*N*=3). Different letters in superscript in each column show statistically significant differences (p<0.05). SL:SC=1:1

Sodium caseinate is a high-molecular-mass protein emulsifier with good surface activity and is able to unfold, adsorb at the oil-water interface and reduce the interfacial tension between the phases. The emulsifying ability of proteins is influenced by the presence of lipophilic amino acids such as phenylalanine, leucine and isoleucine (*26*). During the emulsification, the lipophilic groups modify their confirmation and penetrate the oil phase, whereas the hydrophilic groups protrude into the aqueous phase. Protein such as sodium caseinate produces a loop structure that causes steric hindrance to the oil droplets, preventing their flocculation and coalescence (*27*). The presence of ionic functional groups in protein molecules also provides electrostatic stabilization, keeping the nanodispersion physically stable. Sodium caseinate reduces the interfacial tension between the oil and water phases less efficiently than soy lecithin because it needs to undergo a conformational change at the interface while the soy lecithin, being a small-molecular-mass emulsifier with a clear partitioning of the hydrophobic and hydrophilic parts, would adsorb rapidly at the interface (*28*). Therefore, during the emulsification, the lycopene droplets may have enough time to merge before being fully coated by the sodium caseinate molecules, resulting in the formation of a single larger entity. Another contributing factor was bridging flocculation, which usually occurs when the sodium caseinate chains adsorb to multiple droplets *via* their extended long loops and tails (*29*).

**Fig. 1:**
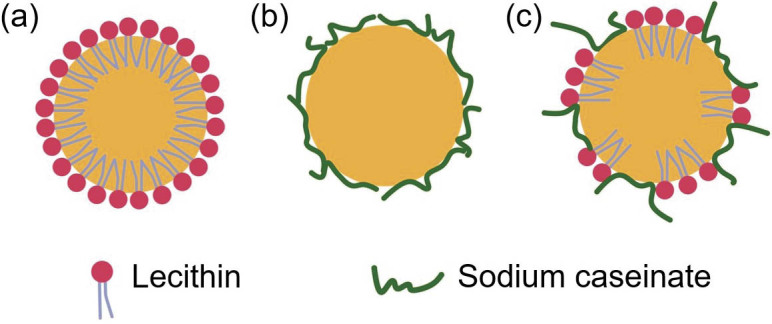
The schematic illustration of lycopene nanodispersion stabilized by: a) soy lecithin, b) sodium caseinate, and c) mixture of soy lecithin and sodium caseinate at 1:1 ratio

The nanodispersion consisting of a mixture of soy lecithin and sodium caseinate showed an intermediate particle size and PDI value, suggesting that both emulsifiers adsorbed at the droplet surface. In the presence of proteins, small molecule surfactants such as phospholipids may interact or compete with the proteins to adsorb at the droplet surface (*30*). During the emulsification, both emulsifiers were expected to adsorb at the surface of the newly created droplets and reduce the interfacial tension between the oil and water phases, preventing them from coalescing. The absorption of the soy lecithin molecules at the droplet surface would affect the absorption mode of the sodium caseinate molecules. With a more limited surface area available on the droplet surface, the adsorbed sodium caseinate molecules would protrude into the aqueous phase rather than elastically spread to cover a maximum area (*31*). This has consequently resulted in steric stabilization, which may prevent the lycopene droplets from aggregating. The soy lecithin molecules will also compete with the sodium caseinate to adsorb at the oil-water interface (*30*) and interact with the adsorbed sodium caseinate to form a strong and densely packed protective layer around the droplet. The adsorption of both emulsifiers on the droplet surface resulted in a combination of electrostatic and steric stabilization, which in turn provided good stability to the emulsion. According to Fang and Dalgleish (*31*), not all the adsorbed proteins are displaced by soy lecithin, suggesting that the surface of the droplets was occupied by the lecithin and sodium caseinate. Small-molecule surfactants, such as lecithin, could also influence the nanodispersion properties by displacing the adsorbed biopolymers from the interface. The ability of the lecithin to displace proteins at the interface was attributable to its small molecular mass, manoeuvrability and strong affinity towards the interface (*32*). It has been reported that, during emulsification with protein as the main emulsifier, the presence of even a small quantity of rapidly adsorbing surfactant can facilitate a substantial reduction in the mean droplet size (*33*). This result shows that a natural emulsifier such as lecithin and a combination of lecithin and sodium caseinate can be used to produce lipid nanodispersion with particle size less than 100 nm, comparable to synthetic surfactants such as Tween 80 and sodium dodecyl sulfate (SDS) (*34*). Fig. 1 shows the schematic illustration of the lycopene nanodispersions stabilized by soy lecithin, sodium caseinate and their mixture.

The influence of emulsifier types on the stability of lycopene nanodispersion against droplet aggregation was determined by measuring the droplet surface charge (zeta potential). Stable lycopene nanodispersion can be produced by using soy lecithin and sodium caseinate separately or in combination, as all samples showed a zeta potential of more than -30 mV (Table 1). A zeta potential value exceeding ±30 mV indicates good stability of a colloidal system. Stabilizing lycopene nanodispersion with soy lecithin resulted in a stable system with a high negative charge (-64 mV). This was due to the presence of negatively-charged phospholipid components, *i.e.* phosphatidylinositol, phosphatidic acid and lysophosphatides. Moreover, the small particle size of the lecithin-stabilized nanodispersion resulted in Brownian effects dominating the gravitational force and thus keeping it stable against aggregation. Although the zeta potential value of the sodium caseinate-stabilized nanodispersion was considerably high, it was the lowest among all samples, presumably due to its tendency to form a network structure. The combination of soy lecithin and sodium caseinate resulted in a significantly lower droplet charge (*i.e*. -48 mV) than soy lecithin alone. The zeta potential was reduced probably due to the depletion flocculation by non-adsorbing sodium caseinate and soy lecithin in the continuous phase. The nanodispersion stabilized with the mixture of soy lecithin and sodium caseinate had a higher zeta potential value than the one stabilized with sodium caseinate alone (*i.e.* -45 mV). Fang and Dalgleish (*31*) found that lecithin can increase the stability of emulsions made from protein.

**Fig. 2:**
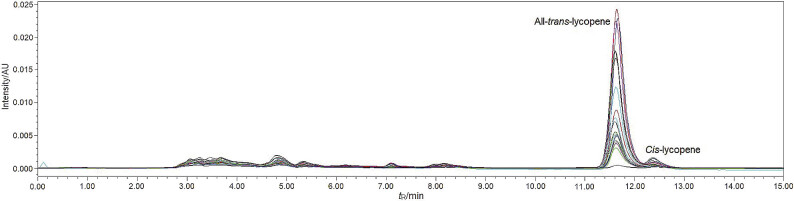
The HPLC chromatograms of lycopene nanodispersions showing peaks of all-*trans-* and *cis*-isomers of lycopene detected at 472 nm

The lycopene concentration in the nanodispersions was quantified using HPLC analysis. Fig. 2 shows the HPLC chromatograms of lycopene in the lycopene nanodispersions prepared in this study. Quantification of the lycopene concentration using HPLC showed that the nanodispersion stabilized with the mixture of soy lecithin and sodium caseinate had the highest lycopene concentration followed by sodium caseinate and finally lecithin (Table 1). It can be postulated that the combination of soy lecithin and sodium caseinate at neutral pH provides better protection to the lycopene against degradation than the soy lecithin and sodium caseinate alone. The enhanced stability of the mixture was ascribed to the interaction between the soy lecithin and sodium caseinate at the interface, as well as the formation of a strong and thick interfacial layer around the droplets (*30*) that prevented lycopene from chemical degradation. The interaction between phospholipids such as lecithin and protein would affect the surface activity, protein structure and net charge of the emulsion (*35*). The sodium caseinate-stabilized nanodispersion had a relatively high lycopene concentration. The sodium caseinate coating layer and extended protein chains from the adsorbed sodium caseinate at the interface generated a strong steric hindrance that protects the droplets against oxidative degradation (*36*). The high stability of lycopene in the nanodispersions stabilized with sodium caseinate and the mixture of soy lecithin and sodium caseinate was also due to the phosphorylated casein peptides that can chelate metal ions (*37*). The sodium caseinate has also been reported to exhibit antioxidative properties, thus minimizing the lycopene loss in the nanodispersion sample (*34*).

### Effect of pH

**Fig. 3:**
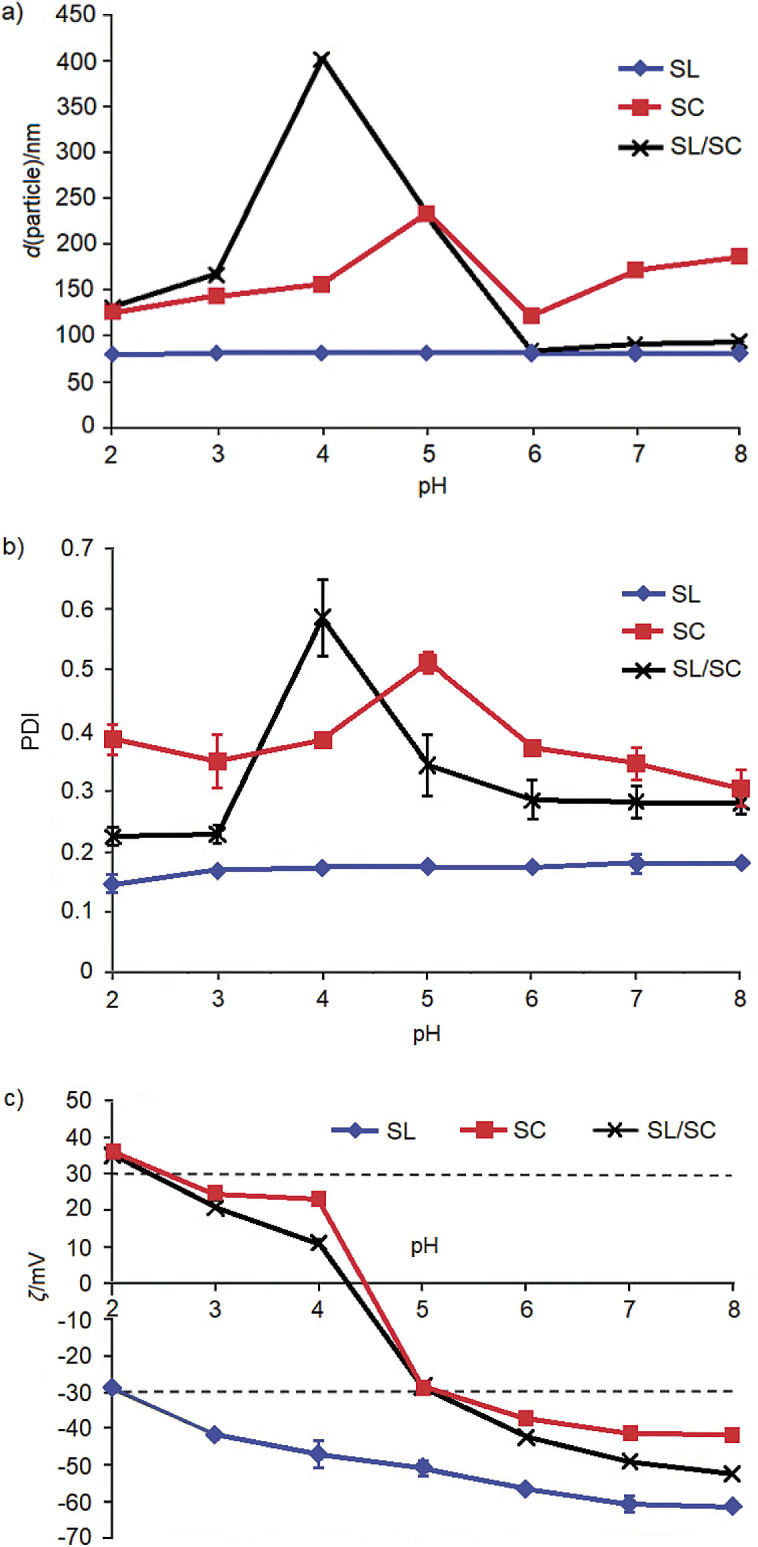
The effect of pH on the: a) particle size, b) polydispersity index (PDI) and c) zeta potential of lycopene nanodispersion stabilized by soy lecithin (SL), sodium caseinate (SC), and soy lecithin and sodium caseinate (SL/SC) in the ratio 1:1

The effects of pH on the particle size, PDI and zeta potential of all lycopene nanodispersions are shown in Fig. 3. Fig. 3a and Fig. 3b show that the soy lecithin-stabilized nanodispersion was highly stable over a wide pH range (pH=2-8), indicating that the soy lecithin layer on the droplet surface was resistant to pH changes and was able to protect the lycopene droplets from aggregating. This is presumably due to the high negative charge carried by phosphatidic acid, phosphatidylinositol and anionic components in soy lecithin that cause the droplets to repulse each other. Other contributing factors are stabilization *via* electrostatic and hydration forces by phosphatidic acid and phosphatidylserine, as well as the high content of surface-active impurities other than phospholipids in lecithin (*38*).

The particle size of sodium caseinate-stabilized nanodispersion decreased with decreasing pH value from pH=8 to pH=6. However, at pH=5, a sharp increase in the particle size was observed, indicating the occurrence of extensive droplet aggregation. Reducing the pH value close to the isoelectric point of sodium caseinate (pH=4-5) resulted in a loss of net charge close to zero. The loss in the surface charge caused the droplets to attract each other because the repulsive force between them was insufficient to overcome the attractive force. When the pH of the nanodispersion was reduced further to pH=2, a significant decrease in the particle size was observed. The reduction in the pH value towards a highly acidic condition resulted in the increased surface charge of sodium caseinate molecules, thus increasing the repulsive force among the coated droplets, preventing droplet aggregation.

Based on the graphs in Fig. 3a and Fig. 3b, the particle size and PDI of the nanodispersion stabilized with the mixture of soy lecithin and sodium caseinate remained small when the pH was reduced from pH=8 to pH=6. At high pH values (pH=6–8), the droplets were highly negatively charged due to the absorption of both sodium caseinate and soy lecithin molecules on the droplet surface. Consequently, the droplets tended to repel each other. In addition, the soy lecithin and sodium caseinate molecules can also interact with each other *via* attraction interaction between the positive patches in the sodium caseinate with the negatively charged soy lecithin components and *vice versa*, thus forming a strong soy lecithin-sodium caseinate complex around the droplet surface that prevents them from aggregating. However, under more acidic conditions (≤pH=5), the nanodispersion became highly unstable, especially at pH=4 and pH=5, which were near the isoelectric point of sodium caseinate. When the pH was reduced further to pH=3 and pH=2, a significant size reduction and reduced PDI value were observed. The sodium caseinate molecules became increasingly positive with increasing acidity. Thus, they were able to interact with negatively charged soy lecithin, which led to an increased repulsion force between the droplets, thus preventing them from aggregating.

The effects of pH on the zeta potential of all nanodispersions are shown in Fig. 3c. Generally, the soy lecithin-stabilized nanodispersion was stable over a wide pH range by exhibiting a high negative net charge exceeding -30 mV at every pH value except at pH=2 (-29 mV). The zeta potential value decreased gradually from pH=8 to pH=2. The reduced negative net charge of the nanodispersion was related to the protonation of the phosphate group of the soy lecithin molecules (*39*).

Nanodispersion made from sodium caseinate and soy lecithin and sodium caseinate showed significant changes in droplet charge (*i.e.*, from highly negative to highly positive when the pH was reduced from pH=8 to pH=2) with a similar trend. This result suggests that sodium caseinate molecules have also been adsorbed on the droplet surface and that not all the adsorbed casein had been displaced by soy lecithin molecules. At pH below the isoelectric point of sodium caseinate, the zeta potential value of soy lecithin and sodium caseinate was slightly lower than that of the sodium caseinate-stabilized nanodispersion, indicating lower stability. The presence of non-adsorbing sodium caseinate in the soy lecithin and sodium caseinate-stabilized nanodispersion (which resulted from the competitive adsorption of soy lecithin molecules and displacement by soy lecithin molecules at the droplet surface) may have resulted in depletion flocculation, hence promoting droplet aggregation (*40*).

### Effect of ionic strength

**Fig. 4:**
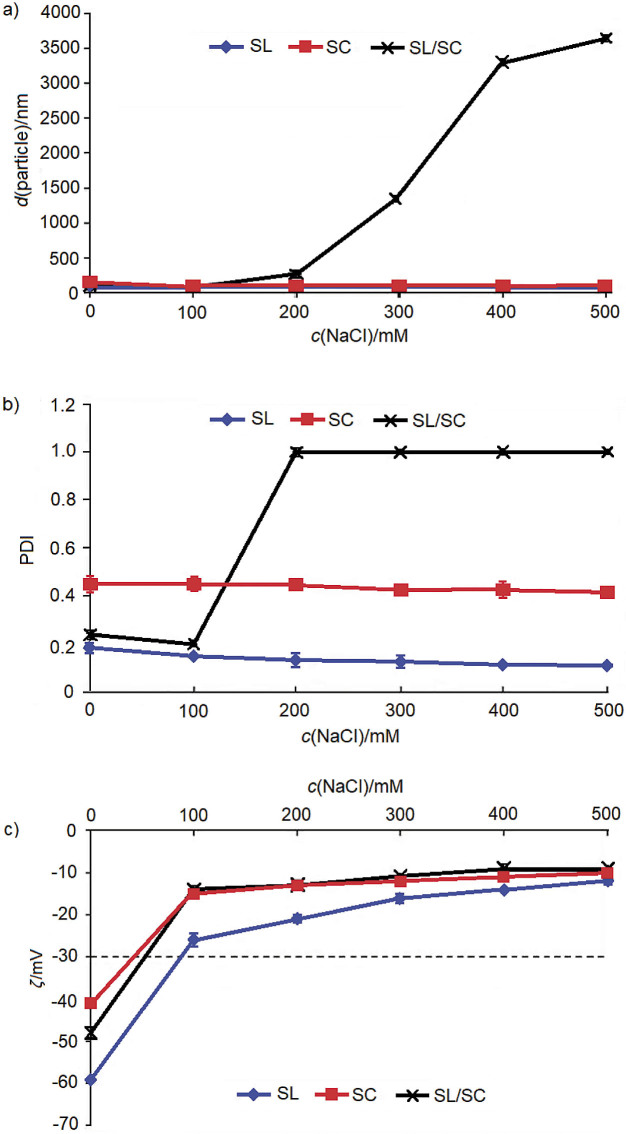
The effect of ionic strength on the: a) particle size, b) polydispersity index (PDI) and c) zeta potential of lycopene nanodispersion stabilized by soy lecithin (SL), sodium caseinate (SC), and soy lecithin and sodium caseinate (SL/SC) in the ratio 1:1

The particle size and PDI of soy lecithin and sodium caseinate nanodispersions were less severely affected by ionic strength than their combination (Fig. 4). The particle size of the soy lecithin-stabilized nanodispersion was small (82 nm) at low NaCl concentrations (≤100 mM), but increased to 108 nm with further increase in the NaCl concentration. An increase in the NaCl concentration beyond 100 mM resulted in the reduction of droplet surface charge due to the electrostatic screening effect (*41*). The increase in the NaCl concentration caused the ionic strength to increase, resulting in reduced droplet charges, hence causing the droplets to enlarge. The reduced curvature of the phospholipid membranes by salt may also promote droplet aggregation (*42*).

The particle size of sodium caseinate-stabilized nanodispersion decreased significantly from 148 nm (without NaCl) to 71 nm (at 100 mM), and it decreased slightly to 66 nm with a further increase in NaCl concentration up to 500 mM. Srinivasan *et al*. (*43*) found that adsorption of α-casein at the droplet surface was enhanced by the addition of NaCl solution up to 40 mM; when 200 mM of NaCl was added, reduced droplet flocculation and improved creaming stability were observed. Dalgleish (*44*) reported that the change in the ionic strength would change casein conformation at the interface, which would in turn affect the steric stabilization.

The particle size of the nanodispersion stabilized with the mixture of soy lecithin and sodium caseinate increased drastically from 91 nm (at 100 mM) to 265 nm (at 200 mM), and it continued to increase up to almost 4 µm (at 500 mM NaCl) (Fig. 4a). The same trend was observed in the PDI result, where the value increased drastically up to 1 when the NaCl concentration exceeded 100 mM, indicating a broad and inhomogeneous size distribution. At low NaCl concentration (≤100 mM), the electrostatic repulsive force was strong enough to overcome the attractive forces between droplets, thus keeping them away from one another. However, beyond 100 mM, the loss in the surface charge of the droplets weakened their repulsive force, thus causing them to be attracted to each other.

The increase in the NaCl concentration resulted in decreased zeta potential (Fig. 4c). Starting from 100 to 500 mM NaCl, all samples showed zeta potential values of less than -30 mV, indicating poor physical stability. Among all samples, nanodispersion stabilized by soy lecithin showed the highest stability at different NaCl concentrations. This may be due to its smaller particle size and narrower size distribution, thus decreasing the tendency to aggregate.

### Effect of temperature

**Fig. 5:**
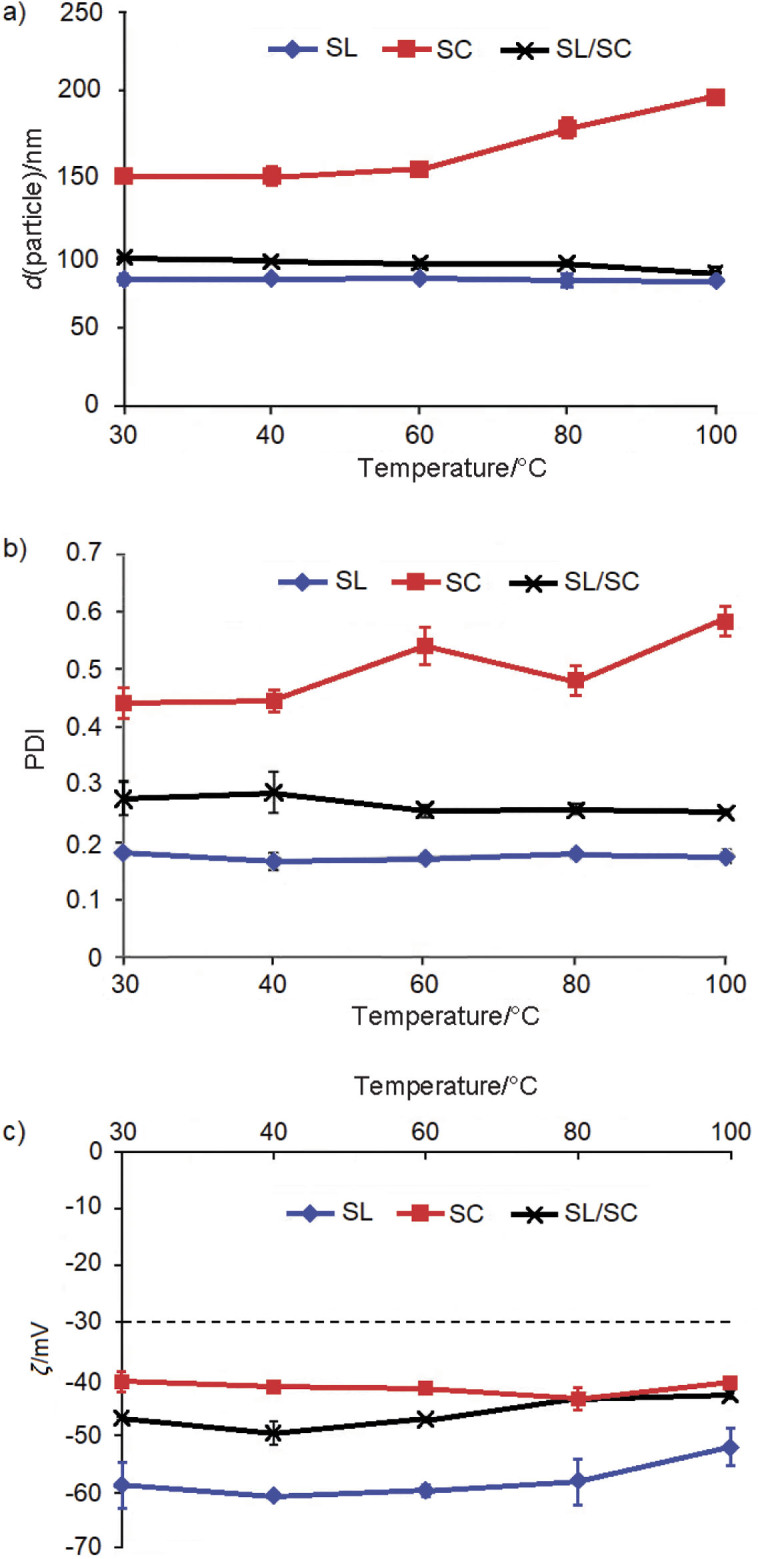
The effect of temperature on the: a) particle size, b) polydispersity index (PDI) and c) zeta potential of lycopene nanodispersion stabilized by soy lecithin (SL), sodium caseinate (SC), and lecithin and sodium caseinate (SL/SC) in the ratio 1:1

The particle size and PDI of the nanodispersions stabilized with soy lecithin and the mixture of soy lecithin and sodium caseinate were remarkably stable over a wide temperature range (Fig. 5). Ozturk *et al*. (*45*) reported that good thermal stability of lecithin resulted from high electrostatic and steric repulsion. In previous research, lecithin was added to milk and combined with other emulsifier types such as whey protein isolate in the emulsion to enhance their thermal stability (*30*). The displacement of sodium caseinate by lecithin at the interface, the formation of a complex of soy lecithin and sodium caseinate, and the increase in the surface charge of casein micelles (*46*) may have contributed to the high thermal stability in the nanodispersion stabilized with the mixture of soy lecithin and sodium caseinate. Additionally, it has been reported that the emulsification activity of a soybean protein-lecithin complex increased after heat treatment (*47*). As for the sodium caseinate-stabilized nanodispersion, the particle size was stable when heated at 30 and 40 °C, but it started to increase gradually with a further increase in temperature. The same trend was observed in the PDI result. The high stability of the sodium caseinate-stabilized nanodispersion at a moderate temperature under neutral pH was related to the crosslinking between the adsorbed sodium caseinate molecules on the same droplet (*48*).

All samples showed good stability at various heating temperatures (30–100 °C) by exhibiting zeta potential values exceeding -30 mV. Comparing the nanodispersions stabilized with sodium caseinate and the mixture of soy lecithin and sodium caseinate, the mixture showed better heat stability than the one stabilized with sodium caseinate alone. The presence of soy lecithin in the emulsion was associated with improved heat stability (*30*).

**Fig. S1:**
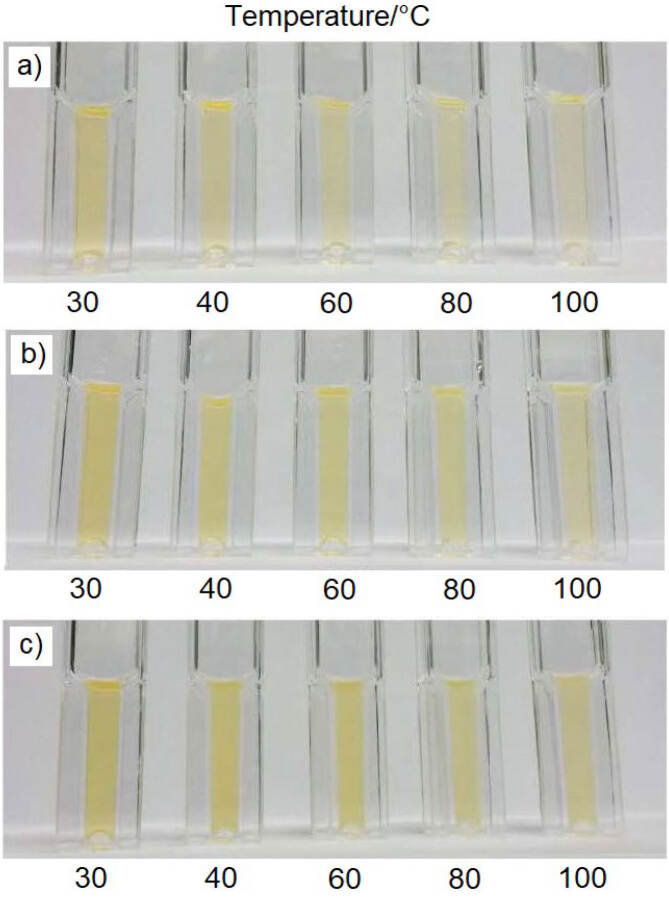
Stability of lycopene nanodispersion stabilized by: a) soy lecithin, b) sodium caseinate, and c) a mixture of soy lecithin and sodium caseinate in the ratio 1:1, incubated at different temperatures for 1 h

The stability of the lycopene nanodispersions against thermal degradation was also investigated by observing the colour intensity of the nanodispersions (Fig. S1), as chemical degradation of carotenoids can be indicated by colour fading (*49*). In Fig. S1, the colour of the soy lecithin-stabilized nanodispersion started to fade at a lower temperature than of the sodium caseinate-containing nanodispersion. The colour fading became more significant at a higher temperature, indicating that the soy lecithin-coated lycopene droplets were susceptible to degradation, particularly at high temperatures. The degradation of the lycopene in the nanodispersion was also accelerated by its small particle size. The colour intensity of the sodium caseinate-stabilized lycopene nanodispersion was higher than of the lecithin-stabilized nanodispersion at all temperatures, indicating better lycopene stability against degradation. This was attributed to the thick stabilizing layer of sodium caseinate at the droplet surface, which hindered lycopene degradation. The nanodispersion stabilized with the mixture of soy lecithin and sodium caseinate was the most stable among all samples. The adsorption of both soy lecithin and sodium caseinate molecules at the same droplet surface resulted in a combination of electrostatic and steric stabilization, thus providing good protection to the lycopene droplets from the chemical degradation.

### *In vitro* bioaccessibility of lycopene nanodispersion

The influence of particle size and emulsifier type on the bioaccessibility of the lycopene nanodispersion was investigated. The particle size of the lycopene nanodispersion was examined just before the samples were subjected to *in vitro* bioaccessibility analysis. Based on the results of particle size in Table 2, the particle size of the lycopene nanodispersions made from different emulsifier types increased in the following order: soy lecithin<mixture of soy lecithin and sodium caseinate<sodium caseinate.

**Table 2:** Particle size and *in vitro* bioaccessibility of lycopene nanodispersion

Sample	*d*(particle)/nm	*γ*(lycopene/(mg/100 mL)			Bioaccessibility/%
Initial sample	Food matrix	Micelles
Soy lecithin (SL)	(77.0±1.2)^a^	(4.6±0.3)^c^	(0.28±0.06)^a^	(0.72±0.01)^c^	(21.9±0.3)^c^
Sodium caseinate (SC)	(156.1±2.4)^b^	(8.2±0.2)^a^	(0.25±0.01)^a^	(1.77±0.01)^b^	(24.7±0.3)^b^
SL/SC	(95.0±0.9)^c^	(7.0±0.4)^b^	(0.26±0.09)^a^	(1.93±0.02)^a^	(31.5±0.1)^a^

The results are expressed as mean value±S.D. (*N*=3). Different letters in superscript in each column show statistically significant differences (p<0.05)

For bioaccessibility, the soy lecithin-stabilized nanodispersion was less bioaccessible than the other two nanodispersions although its initial particle size was smaller (77 nm). Theoretically, smaller particles are more bioaccessible due to the increased surface area that is available for enzyme adsorption. However, an apparent aggregation was observed in the digesta containing the soy lecithin-stabilized nanodispersion when the pH was increased to pH=4 during the first half of gastric digestion. The aggregation decreased significantly when the pH of the digesta was reduced to pH=2 in the second half of the gastric digestion and then increased at pH=6.9 in the small intestinal digestion. The changes in the particle size and structure of the nanodispersion might have reduced its bioaccessibility. The changes in the pH of the digesta alter the electrical charge of the emulsifier used to coat the lipid and consequently cause the structure, interaction and susceptibility to aggregation to change (*50*, *51*). Adjusting the pH digesta to pH=4 might have reduced the zeta potential of the soy lecithin-stabilized nanodispersion, thus promoting droplet flocculation by reducing the repulsion force between droplets. Aggregation may have prevented the lycopene droplets from being easily accessible to the enzymes, leaving the lycopene droplets undigested or minimally digested.

Based on visual observation, no significant aggregation was observed in the digesta containing the nanodispersions stabilized with sodium caseinate or the mixture of soy lecithin and sodium caseinate. Therefore, the initial particle size value of these samples was considered when justifying the bioaccessibility. The lycopene nanodispersion made from the mixture of soy lecithin and sodium caseinate showed the greatest bioaccessibility. This result was ascribed to its smaller particle size. As previously noted, a lycopene nanodispersion with a small particle size provides more surface area for enzyme adsorption, thus enhancing enzyme access to the lycopene droplet or accelerating any chemical reactions occurring at the oil-water interface (*50*). Smaller particle size has been associated with higher solubility, higher micellization rates (increased transfer rate into the bile salt micelles) (*52*), and higher diffusivity rates (enhanced release from the food matrix). Kipp (*53*) claimed that a significant improvement in the bioavailability of a nanoparticle can be expected when the size of the particle is below 100 nm. Additionally, the bioaccessibility of the lycopene nanodispersion was also influenced by the soy lecithin and sodium caseinate molecules at the droplet surface. In the gastric digestion phase, the sodium caseinate molecules might have been extensively digested by pepsin, while the soy lecithin molecules left at the lycopene droplet surface may protect it from chemical degradation and aggregation (*via* electrostatic repulsion). In the subsequent small intestinal phase, the lycopene nanodispersion was continually digested by pancreatin in the presence of bile salts. The sodium caseinate molecules were preferentially digested by proteases, and soy lecithin by phospholipase. This consequently facilitated the release of lycopene into the digesta and increased the amount of lycopene incorporated into the bile salt micelles.

The lower bioaccessibility of the sodium caseinate-stabilized nanodispersion than of the nanodispersion stabilized with the mixture of soy lecithin and sodium caseinate was particularly related to its larger particle size. Although the sodium caseinate molecules at the droplet surface might have been degraded extensively by pepsin and proteases, the digestion process might have been less effective due to the smaller surface area available for adsorption.

## CONCLUSIONS

This study showed that lycopene nanodispersion with nano-sized particles (d<200 nm), good distribution (polydispersity index (PDI)<1), and physically stable (zeta potential ≥30 mV) can be produced with soy lecithin and sodium caseinate, either as the sole stabilizer or in combination at 1:1 ratio, using the emulsification-evaporation method. Under neutral pH conditions, the nanodispersion stabilized by soy lecithin was the most physically stable with the smallest particle size, the lowest PDI and the highest zeta potential value, but it was the least stable against lycopene degradation. To overcome the lycopene degradation issue in the soy lecithin-based nanodispersion, combining it with sodium caseinate at 1:1 ratio was not only able to produce lycopene nanodispersion with small particle size but also to enhance the lycopene stability against degradation. Under different environmental conditions, the soy lecithin-stabilized nanodispersion was not appreciably affected by the changes in the pH, ionic strength and temperature, enhancing its potential to be used in various food systems at wide pH, ionic strength and temperature ranges. Although combining the soy lecithin with sodium caseinate enabled the obtaining of stable lycopene nanodispersion, its application in the food systems with high ionic strength may cause physical destabilization leading to droplet coalescence. The particle size of sodium caseinate-stabilized nanodispersion on the other hand decreased with increasing ionic strength, but changing the pH of the nanodispersion near the isoelectric point of sodium caseinate (pH=4-5) and heating above 60 °C caused the particle size to increase. The presence of sodium caseinate seemed to contribute to the physical instability of the nanodispersion stabilized with the mixture of soy lecithin and sodium caseinate at pH=4-5 but enhanced the *in vitro* bioaccessibility of the lycopene nanodispersion. The lycopene nanodispersion bioaccessibility was not only affected by the particle size, but also the emulsifier types used to prepare the samples. The *in vit*ro bioaccessibility of lycopene nanodispersion increased in the following order: soy lecithin<sodium caseinate<mixture of soy lecithin and sodium caseinate. The information obtained from this study not only provides a better understanding of lycopene nanodispersion physicochemical properties, stability and bioaccessibility in response to various factors, but also the potential emulsifiers for fabricating a delivery system for poorly water-soluble functional lipids.
